# Induction of a Feed Forward Pro-Apoptotic Mechanistic Loop by Nitric Oxide in a Human Breast Cancer Model

**DOI:** 10.1371/journal.pone.0070593

**Published:** 2013-08-12

**Authors:** Suvajit Sen, Brian Kawahara, Jon Fukuto, Gautam Chaudhuri

**Affiliations:** 1 Department of Obstetrics and Gynecology, David Geffen School of Medicine at University of California at Los Angeles, Los Angeles, California, United States of America; 2 Department of Chemistry, Sonoma State University, Rohnert Park, California, United States of America; 3 Department of Molecular and Medical Pharmacology, David Geffen School of Medicine at University of California at Los Angeles, Los Angeles, California, United States of America; 4 Johnson Comprehensive Cancer Center, Los Angeles, California, United States of America; National University of Ireland Galway, Ireland

## Abstract

We have previously demonstrated that relatively high concentrations of NO [Nitric Oxide] as produced by activated macrophages induced apoptosis in the human breast cancer cell line, MDA-MB-468. More recently, we also demonstrated the importance of endogenous H_2_O_2_ in the regulation of growth in human breast cancer cells. In the present study we assessed the interplay between exogenously administered NO and the endogenously produced reactive oxygen species [ROS] in human breast cancer cells and evaluated the mechanism[s] in the induction of apoptosis. To this end we identified a novel mechanism by which NO down regulated endogenous hydrogen peroxide [H_2_O_2_] formation via the down-regulation of superoxide [O_2_^.−^] and the activation of catalase. We further demonstrated the existence of a feed forward mechanistic loop involving protein phosphatase 2A [PP2A] and its downstream substrate FOXO1 in the induction of apoptosis and the synthesis of catalase. We utilized gene silencing of PP2A, FOXO1 and catalase to assess their relative importance and key roles in NO mediated apoptosis. This study provides the potential for a therapeutic approach in treating breast cancer by targeted delivery of NO where NO donors and activators of downstream players could initiate a self sustaining apoptotic cascade in breast cancer cells.

## Introduction

We have previously demonstrated that relatively high concentrations of NO [1 mM DETA-NONOate also referred to as DETA], as released from activated macrophages, induced apoptosis in MDA-MB-468 human breast cancer [HBC] cells by caspase-3 activation [1].These effects were found to be cyclic GMP [cGMP] independent [1].This prompted us to investigate the cGMP independent mechanism[s] of NO action in the apoptosis of HBC cells. In a subsequent and a more recent publication we have also demonstrated that HBC cells strategically maintained significantly higher endogenous levels of H_2_O_2_ compared to normal human breast epithelial [HBE] cells [2]. This was due to the increased production of O_2_^.−^ by HBC cells thereby inhibiting the enzyme activity of catalase resulting in increased intracellular H_2_O_2_ levels [2]. This was evidenced by the elevation of catalase activity in HBC cells following treatment with pegylated superoxide dismutase [PEG-SOD] [2]. Following silencing of catalase activity, there was further increase in H_2_O_2_ levels and this induced HBC growth via the inactivation of PP2A activity [2]. Conversely the reduction of H_2_O_2_ levels in HBC cells following overexpression of catalase, was associated with increased PP2A activity leading to apoptosis [2].

In this study we have demonstrated that one of the cellular mechanisms by which NO induced apoptosis in HBC cells was by decreasing the endogenous levels of H_2_O_2_. NO mediated decrease in H_2_O_2_ levels was associated with both, decreased levels of O_2_^.−^ [a direct precursor of H_2_O_2_], as well as by the induction of the transcription of catalase [an enzyme which metabolizes H_2_O_2_]. We also observed that protein phosphatase 2A [PP2A], and one of its down stream substrates, FOXO1 [a member of the Fork Head Family of transcription factors], played an important role in NO mediated apoptosis. PP2A, an important serine/threonine phosphatase, has been reported to be involved in the regulation of cell homeostasis, through the negative regulation of signaling pathways initiated by kinases [3]. Furthermore, PP2A has been shown to increase the expression of pro-apoptotic proteins such as BIM, and BAX [4]. We have previously reported that reduction of H_2_O_2_ levels [by the overexpression of catalase] could activate PP2A in HBC cells [2]. On this basis we hypothesized that NO may activate PP2A activity via the lowering of H_2_O_2_ levels thereby leading to apoptosis. FOXO1, which has also been reported to induce the synthesis of pro-apoptotic proteins such as BIM and BAX [5], has been shown to be activated by PP2A [5]. Interestingly, we have previously reported that the pro-apoptotic protein BAX, played an important role in the induction of NO mediated apoptosis in MDA-MB-468, HBC cells [1]. We therefore extended our hypothesis to include FOXO1 as an intermediate in the mechanism of NO mediated apoptosis in MDA-MB-468 cells.

In summary, the findings from this study helped identify another novel mechanism by which NO, by modulating endogenous levels of H_2_O_2_ induced apoptosis in HBC cells. The results from this study also underlined the important role of the PP2A-FOXO1 signaling cascade in NO mediated apoptosis.

## Materials and Methods

### Materials

Protease Inhibitor Cocktail [P8430], Catalase-polyethylene glycol [PEG-CAT] [C4963], Superoxide Dismutase-polyethylene glycol [PEG-SOD] [S9549], 3% H_2_O_2_ [#323381], MnTBAP[Mn[III] tetrakis [4-Benzoic acid] porphyrin chloride] 98% pure, was purchased from Calbiochem, SanDiego, CA,. PMSF [P-7626] and all other chemicals including dithiothreitol [DTT], were purchased from Sigma [St. Louis, MO] unless stated otherwise. Hydroethidium [HE] [D11347] was purchased from Molecular Probes [Eugene, OR]. Amplex Red, H_2_O_2_ assay kit # A22188 was purchased from Life Technologies, Carlsbad, CA. The following primary antibodies were used: Catalase [C0979] [Sigma, St. Louis, MO]; PP2A-Cα [sc-130237] and GAPDH [sc-166545] from Santa Cruz Biotechnology, Santa Cruz, CA, Antibodies against FOXO1 [#2880] and pFOXO1[ serine 256][#9461] were obtained from Cell signaling, Danvers, MA. Nitrated BSA [control for nitro tyrosine detection], was obtained from Cayman Chemicals # 89542, Ann Arbor MI. Human breast tissues, normal and from patients with adenocarcinoma were obtained from the translational pathology core laboratory at UCLA, Los Angeles in accordance with university guide lines.

### Methods

#### Cell Culture

Human breast cancer cell lines MDA-MB-468 [ER-VE] [passage no 355] and MDA-MB-231 [ER-VE] [passage No. 350] and MCF-7 [ER+VE] [passage No. 67] were obtained from American Type Culture Collection [Manassas, VA]. DMEM, 1× was purchased from Mediatech [Manassas, VA] [Cat. No. 10-017-CV]. MDA-MB-468 and MDA-MB-231 were grown in DMEM supplemented with 10 mM nonessential amino acids, 2 mM L-glutamine, 1 µg/mL insulin, and 5% fetal bovine serum [FBS]. MCF-7 cells were grown in DMEM supplemented with 10 mM nonessential amino acids, 2 mM L-glutamine, 1 µg/mL insulin, and 5% fetal bovine serum [FBS]. Treatment with DETA-NONOate 1 mM or heat-inactivated controls were done in the presence of serum for 24 h unless specified. Pegylated catalase or pegylated superoxide dismutase treatments were also performed for 24 h for relevant experiments. Following treatment cells were harvested utilizing trypsin and processed for down stream assays. Cells were passaged no more than 10 times after being procured from the company and their genetic characteristics were tested regularly. We regularly checked for the presence of mycoplasma with Lonza's MycoAlert mycoplasma detection kit [LT07-318]. For experimental purposes, cells were allowed to seed overnight prior to all treatments. All treatments were done under serum-free conditions unless otherwise noted.

#### Superoxide detection

Superoxide was determined as described by Zeolonka et.al, [6] which quantifies 2-OH-Ethidium [a specific product of Hydroethidium [HE] and O_2_^.−^] following its separation from other oxidized products of HE by HPLC. Values were normalized against total protein. Briefly 10 µM HE was added in cell culture for 20 m at 37°C. This was followed by lysing in a 0.25-mL lysis buffer [DPBS with 0.1% Triton X-100, pH 7.4], and cell protein levels were measured. A 10-µl lysis solution was used for measuring protein concentration. The remaining solution was mixed with 0.5 mL of 1-butanol, vortex-mixed for 1 min, and centrifuged. The butanol phase was separated and dried with nitrogen. Dried samples were taken up into the solution by adding 0.1 mL of water for HPLC analysis. The cell number was normalized to protein levels in cell lysates, and HPLC peak areas in individual experiments were normalized to the protein concentration.

#### Hydrogen peroxide detection

The rate of H_2_O_2_ production was determined using the Amplex Red Hydrogen Peroxide Assay Kit [Invitrogen, A22188] as per the manufacturer's protocol and as described earlier by Sen et. al [2]. Briefly 50,000 cells were added to 100 µl of an enzyme assay buffer [50 mM potassium phosphate buffer, pH 7.4] containing 0.1 U/mL of horse radish peroxidase [HRP] and 50 µM of Amplex Red. The conversion of Amplex Red to resorufin [Ex/Em: 570/585 nm] was measured over time. The maximum fluorescence intensity was achieved at 4–6 hours after exposure to the Amplex Red reagent.

#### PP2A assay

PP2A activity was assayed using the PP2A Immunoprecipitation Assay Kit [Millipore, 17-313] as per the manufacturer's protocol. Cells [5×10^6^] were scraped from dishes with 0.3 mL phosphate extraction buffer [20 mM imidazole-HCl, 2 mM EDTA, 2 mM EGTA, pH 7.0, with 10 µg/mL each of aprotinin, leupeptin, antipain, soybean trypsin inhibitor, 1 mM benzamidine and 1 mM PMSF. Cells were sonicated for ten seconds, then centrifuged at 2000× g for 5 minutes. After immunoprecipitation of the cellular lysate for PP2A, the enzymatic activity of PP2A was determined by the dephosphorylation of the substrate K-R-pT-I-R-R and the coordination of the resulting free phosphate with Malachite Green. Activity levels were normalized to the amount of PP2A immunoprecipitated purified as determined by SDS-PAGE and Western analysis as previously described. The specific PP2A activities were compared between cell lines and reported as fold-differences. Tissue lysates for PP2A assay were prepared in PP2A assay buffer in the presence of protease cocktail inhibitors using a hand held tissue homogenizer. Extracts were clarified by centrifugation at 12000 g for 30 m just before assaying. Tissues were obtained from the UCLA pathology core facilities which operate within the frame work of UCLA certified protocols to obtain human samples. We did not access any patient information.

#### Western blotting

Cytoplasmic extracts were prepared from cells after various treatments by lysis in buffer containing 50 mm HEPES [pH 7.5], 1 mm DTT, 150 mm NaCl, 1 mm EDTA, 0.1% Tween 20, 10% glycerol, 10 mm β-glycerophosphate, 1 mm NaF, 0.1 mm orthovanadate, 10 µg/ml leupeptin, 10 µg/ml aprotinin, and 0.1 mm phenylmethylsulfonyl fluoride and kept at 4°C for 30 min. cytosolic protein concentrations were quantified by Bradford assay. Equal amounts [20 µgs of protein] were separated on a 10% SDS-PAGE gel and transferred onto polyvinylidene difluoride membranes. Incubation with primary and horse radish peroxidase conjugated secondary antibodies were done at 4°C overnight and 1 h at RT, in 1: 1000 and 1: 10,000 dilutions respectively. Immunoreactivity was detected by using enhanced chemiluminescence [Amersham Biosciences].

#### Detection of apoptosis by flow cytometry

Flow cytometry experiments assaying apoptosis/necrosis were determined with apoptosis/necrosis detection kits containing Annexin V-FITC [BD Pharmingen, 51-6710AK]. Briefly, 1×10^6^ cells were washed twice with cold, 1× PBS and resuspended in 100 µl of 1× Binding Buffer. The cells were transferred to 5-mL culture tubes and stained with 5 µl Annexin V-[Cy3/FITC] and 5 µl of 7-AAD/propidium iodide respectively, as per respective manufacturer's protocol. The cells were incubated for 15 min at room temperature in the dark. 400 µl additional 1× Binding Buffer was added to the samples and the cells were analyzed with a BD FACScan software from Becton Dickinson [Franklin Lakes, NJ]. Apoptotic cells showed an increased staining with Annexin V–FITC wherever applicable.

#### Caspase-3 assay

Control and treated cells were lysed in insect cell lysis buffer [50 mM 4-[2-hydroxyethyl]-1-piperazineethanesulfonic acid, 100 mM NaCl, 2 mM EDTA, 0.1% 3-[[3-cholamidopropyl] dimethylammonio]-1-propanesulfonic acid, 10% sucrose, 5 mM DTT, and 1× protease inhibitor cocktail] for 30 min at 4°C. The lysates were incubated in assay buffer with 20 µg of caspase-3 substrate [acetyl-Asp-Glu-Val-Asp-aminomethylcoumarin] obtained from BD Pharmigen [Franklin Lakes, NJ]. Active caspase-3 activity was monitored by production of fluorescent AMC [Ex/Em: 380/440 nm], which was quantified using a VersaFluor fluorometer from Bio-Rad [Hercules, CA] and operated as per the manufacturer's protocol.

#### Catalase Assay

Catalase assay was performed according to the methods of Beers et.al [7]. Briefly 100 µgs of cell extract was prepared in phosphate buffer pH 7.0 and mixed with 1 mM H_2_O_2_ at 37°C in a quartz cuvette and the disappearance of H_2_O_2_ was measured over time at 240 nm. Results were normalized as difference in final versus initial absorbance per unit time per unit µgs of protein extract.

#### Quantitative PCR

Total RNA from cells was purified using TRIzol reagent [Invitrogen] according to the manufacturer's protocol, and cDNA was synthesized with oligo[dT] primers using SuperScript III [Invitrogen]. Real-time RT-PCR analysis was carried out with iQ SYBR Green supermix [Bio-Rad] on an iCycler iQ real-time PCR detection system for forty cycles [Bio-Rad]. β-actin [human] was used as an internal control to calculate the relative expression. Primer pairs were obtained from Qiagen for human β-actin [PPH000420b-200], human catalase [PPH00420B-200], and human FOXO1 [PPH01964F-200], and from Santa Cruz Biotechnology for the catalytic subunit of human PP2A [sc-43509-PR]. In each case, relative mRNA expression values [Target mRNA vs β-actin] were calculated using the 2^−[ΔΔCt]^ method.

#### Gene silencing utilizing siRNA

SiRNAs againt the PP2A catalytic subunit [NM _002715] and FOXO1 [NM_002015.3] were obtained from Santa Cruz biotech, PP2A Cα siRNA [Sc-43509] and FOXO1siRNA [Sc-35383] respectively were obtained from Santa Cruz Biotech Non-specific, scrambled siRNA from the same company were used as a control. siRNA against human catalase was also obtained from Santa Cruz biotech # Sc 45330. Briefly 2×10^5^ cells were seeded in a 6-well plate one day prior to transfection. For each well, the siRNA stock was diluted in the appropriate amount of Opti-MEM I reduced serum medium and the cells were transfected with LipofectAMINE™ 2000 Invitrogen, CA, USA], according to the manufacturer's instructions. Cells were incubated at 37°C in a CO_2_ incubator for 24–72 h and the effect of gene silencing was analysed by immunoblots. Appropriate cell lysates from Santa Cruz Biotech, Sc-80665, and Sc-2221 were used as controls for western blots for PP2Aand FOXO1 respectively.

#### Statistics

All values are expressed as mean ± SE. Each value is the mean of at least three separate experiments in each group. The differences in the effects of drug treatment when compared with control values were analyzed by students T test. *P* values equal to or less than 0.05 were considered significant, **<p.005 *<p.05.

## Results

### DETA treatment modulated the ROS status in MDA-MB-468 cells

**Figure 1:**
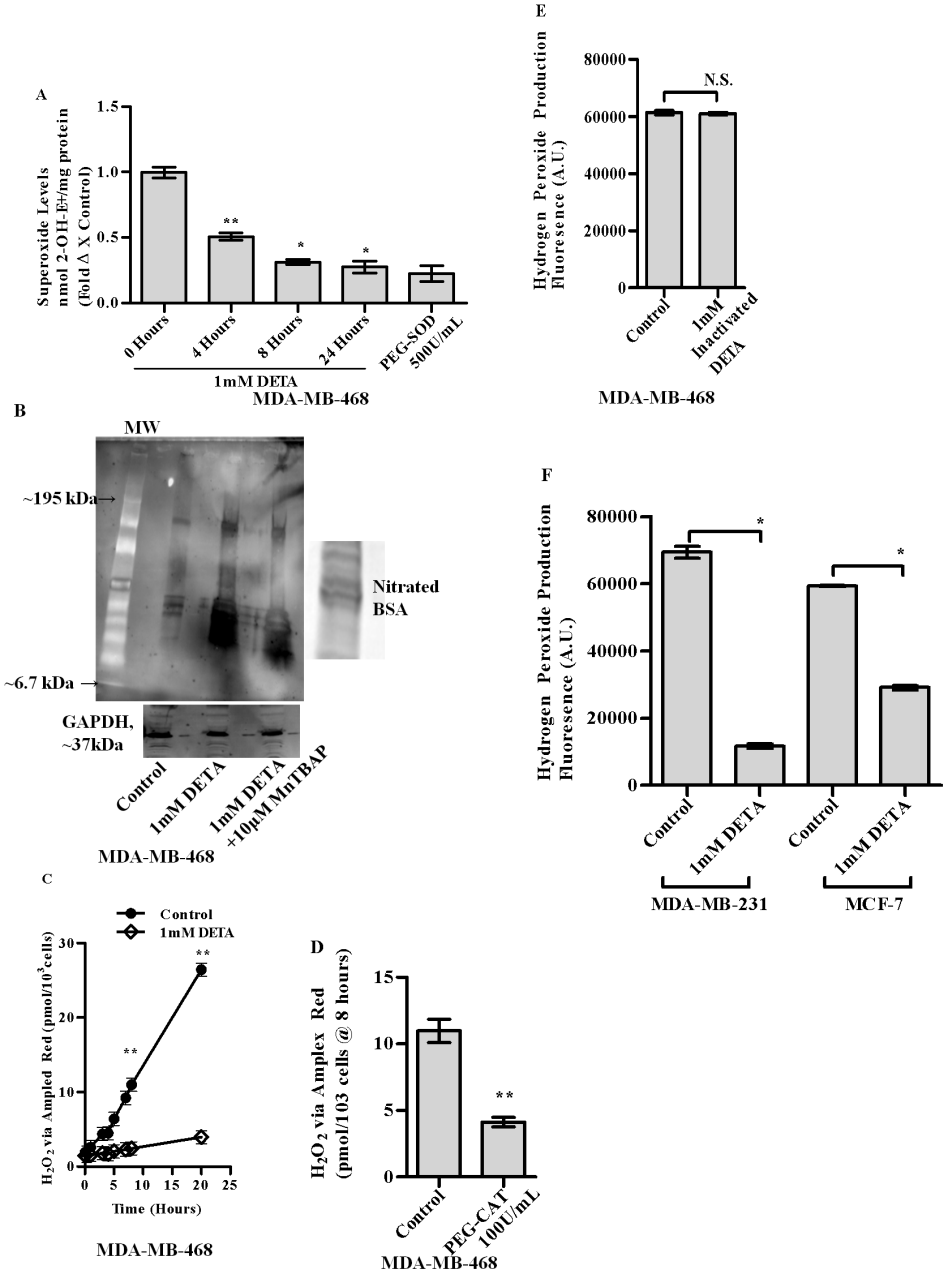
Fold changes in O_2_^.−^ levels in MDA-MB-468 upon treatment with 1 mM DETA-NONOate over 24 h [a]. Western blot analysis of MDA-MB-468 cell lysate using anti-3-nitrotyrosine antibody at 24 h following treatment with 1 mM DETA-NONOate, with or without prior treatment with 10 µM MnTBAB [b]. Rate of H_2_O_2_ production as measured by Amplex Red in MDA-MB-468 cells with or without the treatment with 1 mM DETA-NONOate [c]. H_2_O_2_ production determined in MDA-MB-468 cells with or without the treatment of pegylated-catalase [100 U/mL] at 8 h [d]. H_2_O_2_ production determined in MDA-MB-468 cells with or without inactivated DETA [e]. H_2_O_2_ production determined in MDA-MB-231 and MCF-7 upon treatment with 1 mM DETA-NONOate at 24 hours [f]. Results are expressed as the means from three independent experiments performed in duplicate; *bars*, SE wherever applicable. Western blot images are representative of three different experiments. GAPDH is probed as an equal loading control wherever applicable.

DETA [1 mM], reduced the endogenous superoxide [O_2_^.−^] levels in MDA-MB-468 cells associated with increased formation of nitrated tyrosine species, which was partially reversible upon pre-incubation with MnTBAP [Fig. 1a & b]. PEG-SOD treatment was used as a control to validate the assay system. 500 U/mL of PEG-SOD or vehicle control [ 0.1% DMSO] was added to cell cultures for 24 h before assaying for O_2_^.−^ production. This was also accompanied by a decrease in the H_2_O_2_ production over time [Fig. 1c]. Pegylated catalase [PEG-CAT] treatment was used as a control to validate the assay system. 100 U/mL of PEG-CAT or vehicle controls [ 0.1% DMSO] were added to cell cultures for 24 h before assaying for H_2_O_2_ production.[Fig. 1d]. Exposure of MDA-MB-468 cells to heat inactivated DETA ie DETA kept at 37°C for 7 days as validated earlier [1] did not affect the endogenous H_2_O_2_ levels confirming that the observed events were specific to the modulations of NO. Two other breast cancer cell lines, MDA-MB-231 [Estrogen receptor –ive] and MCF7 [Estrogen receptor +ive] also exhibited a decrease in the endogenous levels of H_2_O_2_ at 24 h following exposure to 1 mM DETA [1f].

### Important role of PP2A in NO induced apoptosis

**Figure 2:**
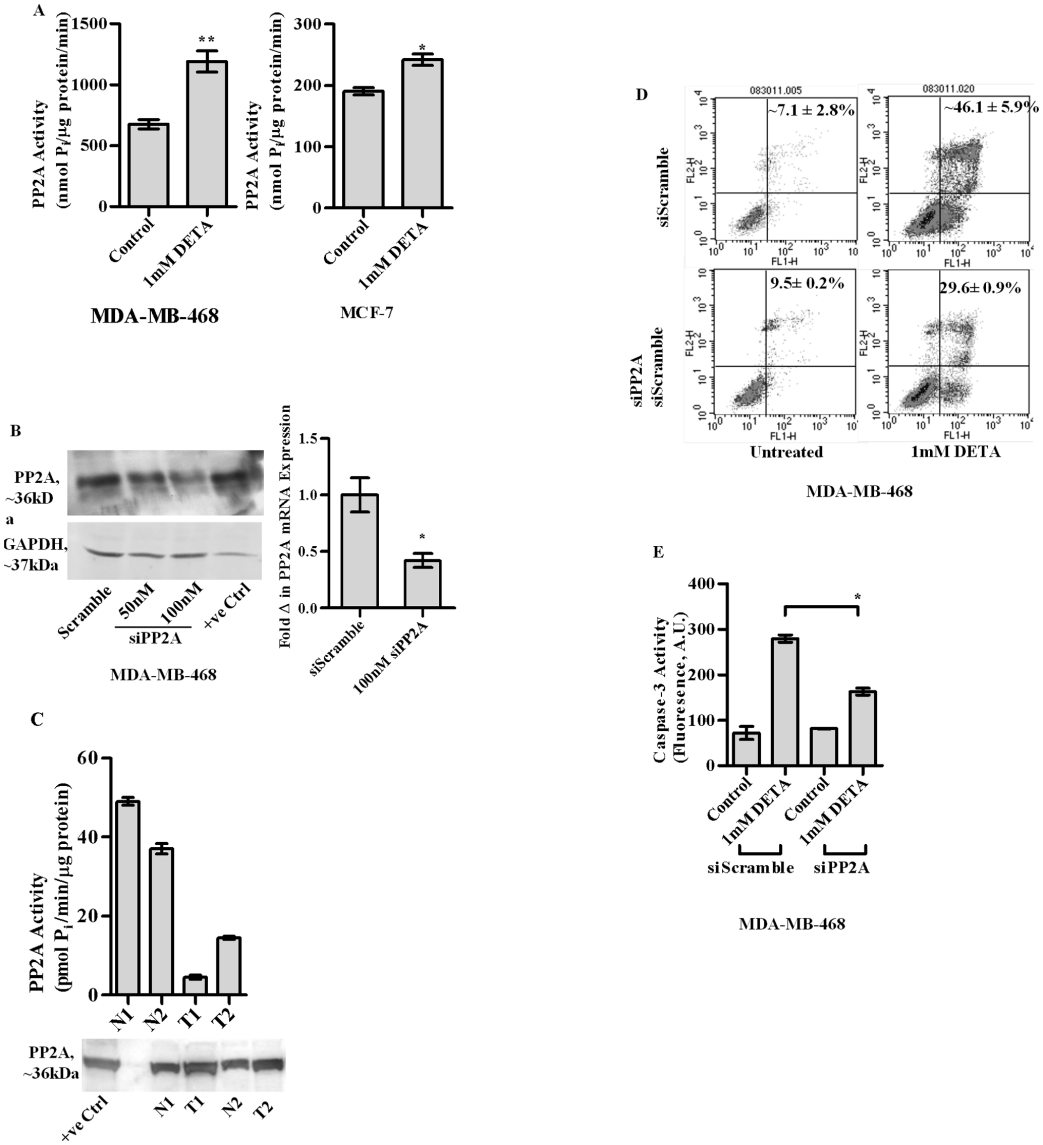
PP2A activity in cell lysates of MDA-MB-468 cells at 24 h following treatment with or without 1 mM DETA-NONOate [a, left panel] PP2A activity in cell lysates of MCF-7 at 24 h following treatment with or without imM DETA-NONOate [a, right panel]. Silencing of the catalytic subunit of human PP2A utilizing siRNA depicted by immunoblotting [b, left panel]. Silencing of the catalytic subunit of human PP2A utilizing siRNA depicted by qPCR [b right panel]. Annexin V binding assay at 24 h, in MDA-MB-468 cells either transfected with scrambled controls [siScramble] or siRNA against the human PP2A gene [siPP2A] [c]. Caspase-3 activity in MDA-MB-468 at 24 h following treatment with 1 mM DETA-NONOate in either scrambled controls [siScramble] or silenced for the catalytic subunit of PP2A [siPP2A] following treatment with 1 mM DETA-NONOate [1 mM DETA] [d]. PP2A activity in two tissue samples from either normal breast or those from patient tumors: denoted by N1, N2, and T1,T2 respectively [e]. Results are expressed as the means from three independent experiments performed in duplicate; *bars*, SE wherever applicable. Western blot images are representative of three different experiments. GAPDH is probed as an equal loading control wherever applicable.

A recent publication from our lab elaborated the inverse correlation between endogenous H_2_O_2_ levels and PP2A activity in breast cancer models [2]. Interestingly following exposure to 1 mM DETA, both MDA-MB-468 and MCF7 breast cancer cells exhibited an increase in PP2A activity. [Fig. 2a, left and right panels respectively]. As PP2A activity has been reported to be associated with increased apoptotic tendency in HBC cells [2], we assessed its role in NO mediated apoptosis. An attenuation in the increase of Annexin V binding and caspase −3 activity was observed upon DETA-NONOate treatment on MDA-MB-468 cells silenced for the catalytic subunit of PP2A [Fig. 2b–2d]. There was a modest increase in Annexin V binding upon PP2A silencing which may be attributed to cell proliferative properties of PP2A [19].

### Important role of FOXO1 in NO induced apoptosis

**Figure 3:**
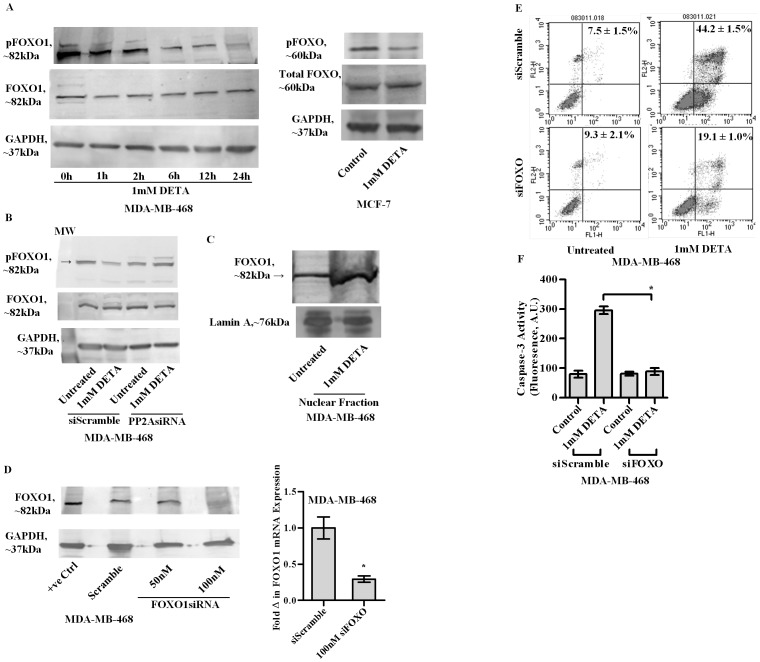
Immunoblot analysis of the dephosphorylation of pFOXO1 at serine 256, in MDA-MB-468 cell lysates, over time following treatment with 1 mM DETA-NONOate [a, left panel]. Immunoblot analysis of the dephosphorylation of pFOXO1 at serine 256 in MCF-7 cell lysates at 24 h post-treatment with 1 mM DETA-NONOate [a right panel]. Immunoblot analysis of dephosphorylation of pFOXO1 upon DETA-NONOate treatment in either scrambled controls or MDA-MB-468 cells silenced for the catalytic subunit of PP2A [PP2A siRNA] [b]. Immunoblot analysis of FOXO1 in MDA-MB-468 nuclear fractions at 24 h post-treatment with 1 mM DETA-NONOate [c ]. Silencing of FOXO1 in MDA-MB-468 cells utilizing siRNA against human FOXO1 [d left panel] qPCR analysis to determine the fold change in mRNA expression of FOXO1 in MDA-MB-468 transfected with either scrambled controls [SiScramble] or siRNA against the human FOXO1 gene [siFOXO] [d, right panel]. Annexin V binding assay at 24 h, in MDA-MB-468 cells either transfected with scrambled controls [siScramble] or siRNA against the human FOXO1 gene, following treatment with 1 mM DETA-NONOate [1 mM DETA] [e]. Caspase-3 activity in MDA-MB-468 at 24 h following treatment with 1 mM DETA-NONOate in either scrambled controls [siScramble] or silenced for human FOXO1 gene [siFOXO] [f]. Results are expressed as the means from three independent experiments performed in duplicate; *bars*, SE wherever applicable. Western blot images are representative of three different experiments. GAPDH and Lamin A are probed as equal loading controls for cytosolic and nuclear nuclear fractions respectively.

FOXO1, an important pro-apoptotic transcription factor has been reported to be a substrate for PP2A [4]. We assessed the role of PP2A-FOXO1 interplay in NO mediated apoptosis in breast cancer. Interestingly it was observed that NO treatment induced the dephosphorylation of pFOXO1 in MDA-MB-468 as well as in MCF7 cells. [Fig. 3a, left and right panels], and prior silencing of PP2A attenuated the dephosphorylation event as exhibited in MDA-MB-468 cells. [Fig. 3b]. Increased nuclear concentration of FOXO was observed at 4 h following DETA treatment in MDA-MB-468 cells [Fig. 3c]. Significant dephosphorylation of pFOXO1 was first observed between 2–6 h [Fig. 3a]. Interestingly significant FOXO accumulation was observed at 4 h [between 2 and 6 h ] following DETA treatment. This is in agreement with the established observation that FOXO translocates to the nucleus following dephosphorylation. Furthermore it was observed that DETA treatment on cells silenced for FOXO1 exhibited decreased Annexin V binding and caspase -3 activity compared with scrambled controls [Fig. 3d–f]. Interestingly siFOXO exhibited increased Annexin V binding compared to siScramble. This could be attributed to the activation of some of its isoforms such as FOXO3 as a compensatory mechanism in response to FOXO1 silencing. Indeed FOXO3 has been observed in breast cancer and does play a role in apoptosis [20]. A modest increase in Annexin V binding in siFOXO following DETA treatment suggests the existence of alternative pathways independent of FOXO1 in NO mediated apoptosis. Thus the PP2A-FOXO1 axis is an important pathway but certainly not the only one.

### Important role of catalase in NO induced apoptosis

**Figure 4:**
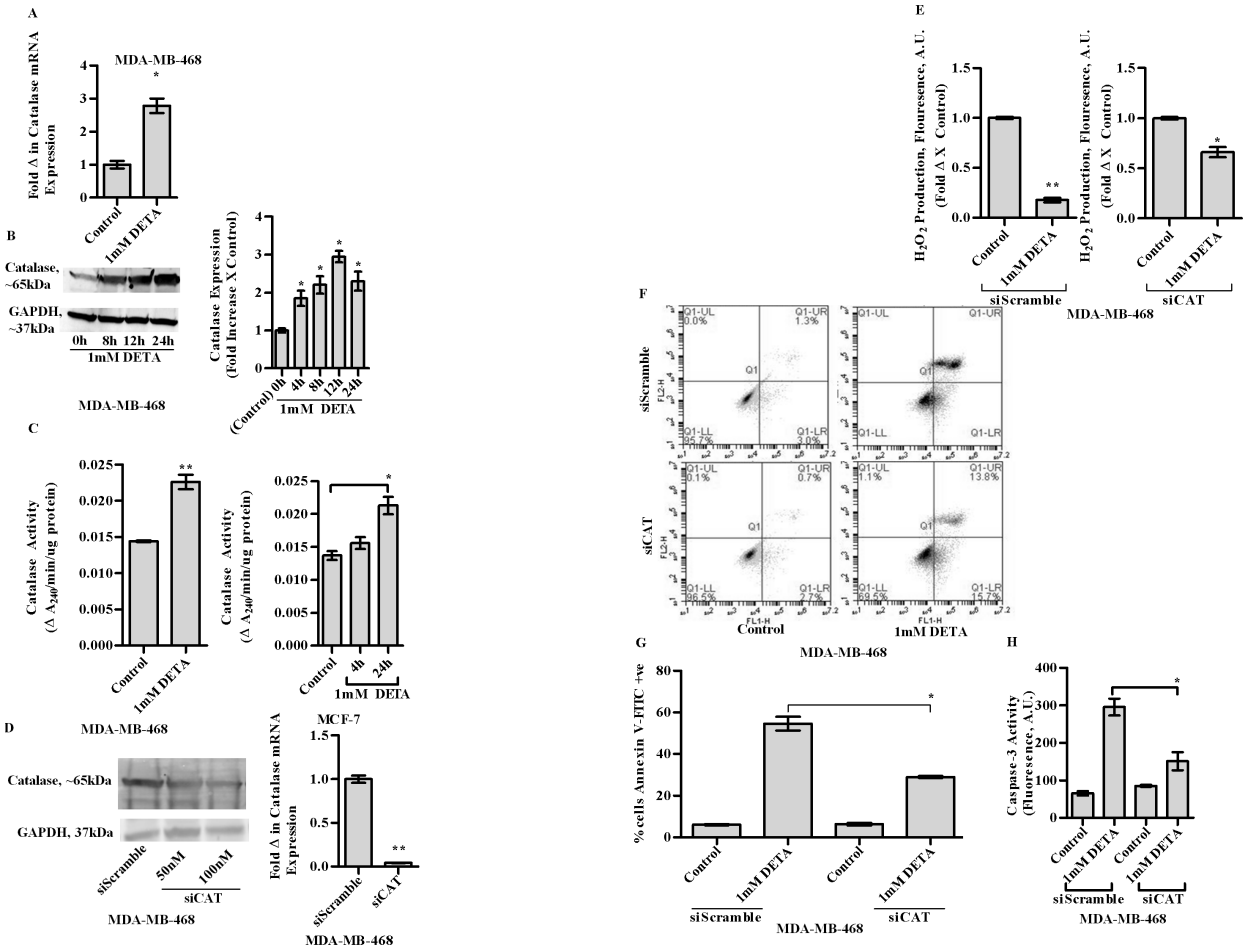
qPCR analysis to determine fold change in mRNA expression of catalase in MDA-MB-468 cells at 4 h following treatment with 1 mM DETA-NONOate [a]. Immnoblot analysis of the expression of catalase in lysates of MDA-MB-468 cells following treatment with 1 mM DETA-NONOate [b, left panel]. Densitometric analysis of catalase expression utilizing the Image Quant software [b, right panel]. Enzyme activity of catalase in 100 µg of cell lysate of MDA-MB-468 cells with or without the addition of 1 mM DETA-NONOate [c, left panel] Enzyme activity of catalase in 100 µg of cell lysate of MCF-7 cells with or without the addition of 1 mM DETA-NONOate [c, right panel]. Silencing of catalase in MDA-MB-468 cells utilizing siRNA against human catalase depicted by immunobloting and qPCR [d, left and right panel]. H_2_O_2_ production in MDA-MB-468 cells either transfected with scramble controls or siRNA against catalase, at 24 h following treatment with 1 mM DETA-NONOate [e]. Annexin V binding assay at 24 h, in MDA-MB-468 cells either transfected with scrambled controls [siScramble] or siRNA against the human catalase gene, following treatment with 1 mM DETA-NONOate [1 mM DETA] [f]. Graphical representation of the quantification of cells undergoing apoptosis in either scramble controls or in catalase silenced MDA-MB-468, following treatment with 1 mM DETA-NONOate [g]. Caspase-3 activity in MDA-MB-468 at 24 h following treatment with 1 mM DETA-NONOate in either scrambled controls [siScramble] or silenced for human catalase gene [siCAT] [h]. Results are expressed as the means from three independent experiments performed in duplicate; *bars*, SE wherever applicable. Western blot images are representative of three different experiments. GAPDH is probed as an equal loading control wherever applicable.

Several reports have described the induction of antioxidant gene expressions [including catalase], upon activation of FOXO1 [8]. Moreover we have shown earlier, that overexpression of catalase led to apoptosis [2]. We therefore investigated the role of catalase in NO mediated apoptosis. Interestingly we observed that, DETA [1 mM] increased the mRNA as well as protein expression of catalase associated with an increase in its enzyme activity [Fig. 4a–c, left panel]. Increase in catalase bioactivity, following DETA treatment was also observed in another breast cancer cell line, MCF7 [Fig. 4c right panel]. Investigation of the critical role of catalase in NO mediated apoptosis utilizing siRNA mediated gene silencing revealed: [1] Attenuation in the decrease in endogenous H_2_O_2_ levels following DETA treatment [Fig. 4e] [2] Attenuation in the increase of Annexin V binding upon DETA treatment [ Fig. 4 f] [3] Attenuation in the decrease in caspase- 3 activity following DETA treatment [Fig. 4g–h].

### Important role of the PP2A and FOXO1 in the induction of catalase gene expression

**Figure 5:**
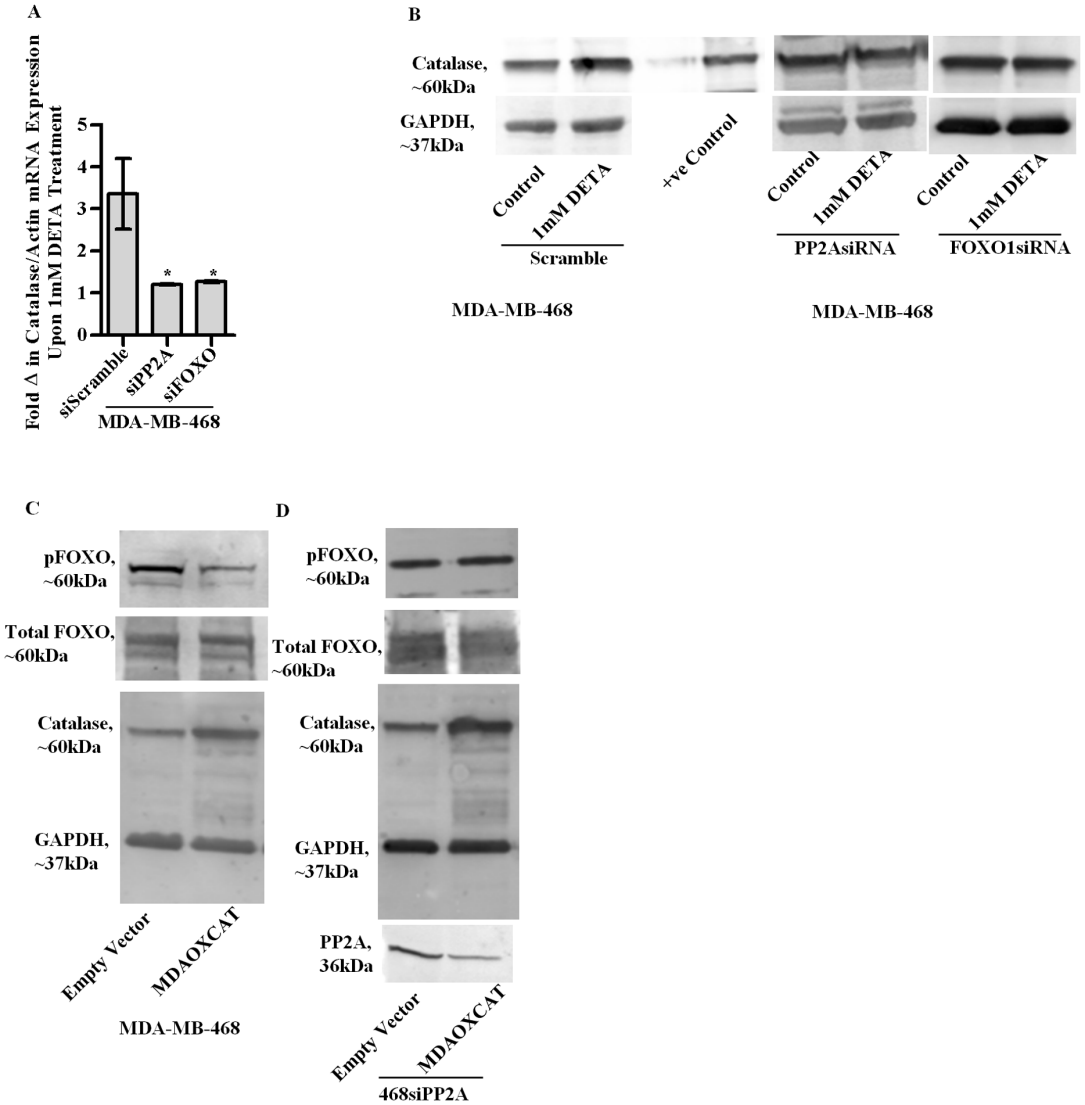
qPCR analysis to compare the expression of catalase mRNA in MDA-MB-468 cells transfected with scrambled controls, siRNA against FOXO1 and SiRNA against PP2A following treatment with 1 mM DETA-NONOate for 4 h [a]. Immunoblot analysis of catalase protein expression in the lysates of MDA-MB-468 cells transfected with scrambled controls, siRNA against FOXO1 and siRNA against PP2A at 24 h following treatment with 1 mM DETA-NONOate [b]. Immunoblot analysis of total and pFOXO1 in lysates of MDA–MB-468 cells at 24 h, following overexpression of either the human catalase gene [MDAOXCAT] or empty vectors in scrambled controls or in cells silenced for the catalytic subunit of PP2A [c–d]. Results are expressed as the means from three independent experiments performed in duplicate; *bars*, SE wherever applicable. Western blot images are representative of three different experiments. GAPDH is probed as an equal loading control wherever applicable.

Investigating the role of PP2A and FOXO1 in the induction of catalase revealed attenuation in the increase of catalase mRNA and protein expression, upon DETA treatment in either PP2A or FOXO1 silenced MDA-MB-468 cells [Fig. 5 a and b]. Interestingly overexpression of catalase [MDAOXCAT] led to the dephosphorylation of pFOXO1 compared to empty vector controls [Empty Vector] indicating NO initiates a feed forward mechanistic loop to cause apoptosis in MDA-MB-468 cells [Fig. 5c]. As expected the dephosphorylation event was inhibited upon PP2A silencing [Fig. 5d] indicating the dependency of the phosphorylation process on PP2A activity.

## Discussion

In this study we have elucidated a novel mechanism by which relatively high concentrations of NO [as released from activated macrophages ie ∼0.5 µM NO over 24 h] [18], modulated the levels of endogenous ROS to induce apoptosis in HBC cells. We have previously demonstrated that NO, in concentrations [as released from activated macrophages and mimicked by the treatment of 1 mM DETA-NONOate], induced apoptosis in MDA-MB-468 HBC cells [1]. We observed that NO induced a pro-apoptotic cascade of events involving the activation of MKP1, a dual specificity phosphatase, which was responsible for the dephosphorylation of ERK1/2, which in-turn was associated with the integration of BAX to the inner mitochondrial membrane to cause apoptosis [1]. More recently we demonstrated the existence of an endogenous ROS mediated, pro-survival mechanism, which was constitutively operational in HBC cells. We observed that HBC cells strategically maintained elevated levels of H_2_O_2_ [when compared to HBE cells], to inhibit PP2A [a serine threonine phosphatase, also known to inactivate ERK1/2 and AKT], to elicit continuous growth. In this study we have elucidated that along with the induction of a pro-apoptotic pathway, NO treatment also initiated an “anti – growth” mechanism in which it directly interfered with the ROS mediated growth signaling to supplement the pro-apoptotic pathway.

Our data indicated that NO treatment significantly decreased H_2_O_2_ levels in HBC cells. This was a consequence of both a reduction in the levels of O_2_^.−^ , a precursor of H_2_O_2_ and the activation of catalase, one its metabolizing enzyme. The decrease in O_2_^.−^ levels suggested that NO, either inhibited the production of O_2_^.−^ or directly quenched it . There are reports of NO inhibiting the assembly of NADPH oxidases to reduce O_2_^.−^ production [9]. This could be a possibility in our model system as well, as HBC cells have been reported to exhibit increased expressions of NADPH-oxidases, such as NOX4 [10]. It would be interesting to investigate the role of NO in the assembly processes of mitochondrial electron chain complexes which are seats of O_2_^.−^ generation. As NO can function as a heme ligand [11], it could be envisaged that it might interfere with the heme insertion processes during the assembly of key heme proteins such as cytochrome c oxidase. As far as the direct quenching of O_2_^.−^ is concerned there could be the possibility of peroxynitrite formation as the rate of combination between the reactants has been reported to be as high as 2×10^10^ [12]. Nonetheless, controversy exists regarding the role of peroxynitrite in various pathophysiological processes.

Our observation that the formation of nitrated tyrosine species, was associated with that of O_2_^.−^ reduction , suggested the involvement of reactive nitrogen species, that caused nitration of tyrosine residues in proteins. This could most likely be nitrogen dioxide, as tyrosine nitration is dioxygen dependent [13]. The exact identification or mechanism of formation of the relevant nitrogen species remained to be investigated. Our observation that the formation of nitrated tyrosine species was partially reversible by MnTBAP may suggest the intermittent formation of peroxynitrite [as there have been reports describing MnTBAP to be a specific quencher of peroxynitrite [14], or MnTBAP could simply have acted as a superoxide dismutase mimetic as indicated by other investigators [15]. Nonetheless, our data did indicate that NO treatment reduced endogenous O_2_^.−^ levels which was associated with the nitration of tyrosine residues. The decrease in O_2_^.−^ levels was associated with a decrease in endogenous H_2_O_2_ levels. This decrease was observed in a panel of breast cancer cell lines and was not observed when cells were exposed to heat inactivated DETA. Thus the attenuation in the endogenous levels of H_2_O_2_ upon exposure to NO was universal and specific to the actions of NO. The decrease in H_2_O_2_ levels could be an effect of both genomic and non genomic actions of NO. The non genomic action could be due to a decrease in the levels of O_2_^.−^ its direct precursor, or an activation of catalase due to an attenuation of its inhibition by O_2_^.−^ as reported earlier [2]. The genomic action could be attributed to the signaling events connecting activation of PP2A , dephosphorylation of FOXO1 and subsequent transcription of catalase.

We have previously demonstrated a direct correlation of PP2A activation with the reduction in the basal levels of H_2_O_2_ in HBC cells [2]. In this study we observed the activation of PP2A in at least two breast cancer cell lines, [MCF7 and MDA-MB-468] following NO treatment. Therefore, NO mediated reduction in H_2_O_2_ levels and associated activation of PP2A further underlines the inverse correlation between the activation of PP2A and [H_2_O_2_] in HBC cells. Interestingly we have observed that tissues from breast tumors exhibited decreased PP2A activity compared to their normal counter parts. This could have resulted from inactivation of the thiols of the PP2A active site by elevated H_2_O_2_ levels observed in cancer cells. Thiol oxidation by H_2_O_2_ at the active site of phosphatases, have been described in several reports [16]. Thus reduced levels of H_2_O_2_ would shift the equilibrium towards more active phosphatases as reported earlier [16].

FOXO1 has been reported to be a pro-apoptotic transcription factor which when activated by dephosphorylation at serine 256 has been shown to transcribe pro-apoptotic genes including BAX and BAD [17]. Our results further indicated that PP2A caused FOXO1 activation, as PP2A silenced cells failed to exhibit the dephosphorylation of pFOXO1 following NO treatment. This indicated that NO activated FOXO1 via PP2A. FOXO1 induces the transcription of antioxidant genes such as catalase and superoxide dismutase along with pro-apoptotic molecules as mentioned earlier [17]. Increased accumulation of FOXO1 in the nuclear fraction following DETA treatment further indicated the important role of FOXO1 in the transcriptional regulation catalase. Our data indicated that FOXO1 silencing attenuated mRNA expression of catalase upon treatment with NO. The same effect on catalase mRNA was observed following silencing of PP2A thereby indicating that the PP2A-FOXO1 mechanistic axis was important in the induction of catalase following treatment with NO. Conversely, overexpression of catalase caused the dephosphorylation of pFOXO1 in scrambled controls but not in MDA-MB-468 cells silenced for PP2A, indicating the operation of a feed forward activation loop in NO mediated apoptosis.

The major events in the mechanism of NO induced apoptosis such as activation of catalase, PP2A and dephosphorylation of phosphorylated FOXO1 were also observed in MCF7 breast cancer cells. In summary, this study provides data that can be exploited therapeutically, in that estrogen coupled with NO donors could be targeted to the breast tissue. Subsequently the NO released, would induce apoptosis specifically in the transformed cells which are known to produce increased levels of ROS. NO would interact with the endogenous ROS to induce apoptosis as suggested by this work.
